# Supplementary material for: Photosynthesis, Respiration, and Growth of Five Benthic Diatom Strains as a Function of Intermixing Processes of Coastal Peatlands with the Baltic Sea
Source: Microorganisms. 2022 Mar 30;10(4):749. doi: 10.3390/microorganisms10040749 (PMC9030513; doi:10.3390/microorganisms10040749)
Supplement: Supplementary file 1 [file microorganisms-10-00749-s001.zip › microorganisms-1645895-Supplementary Figures.pdf]

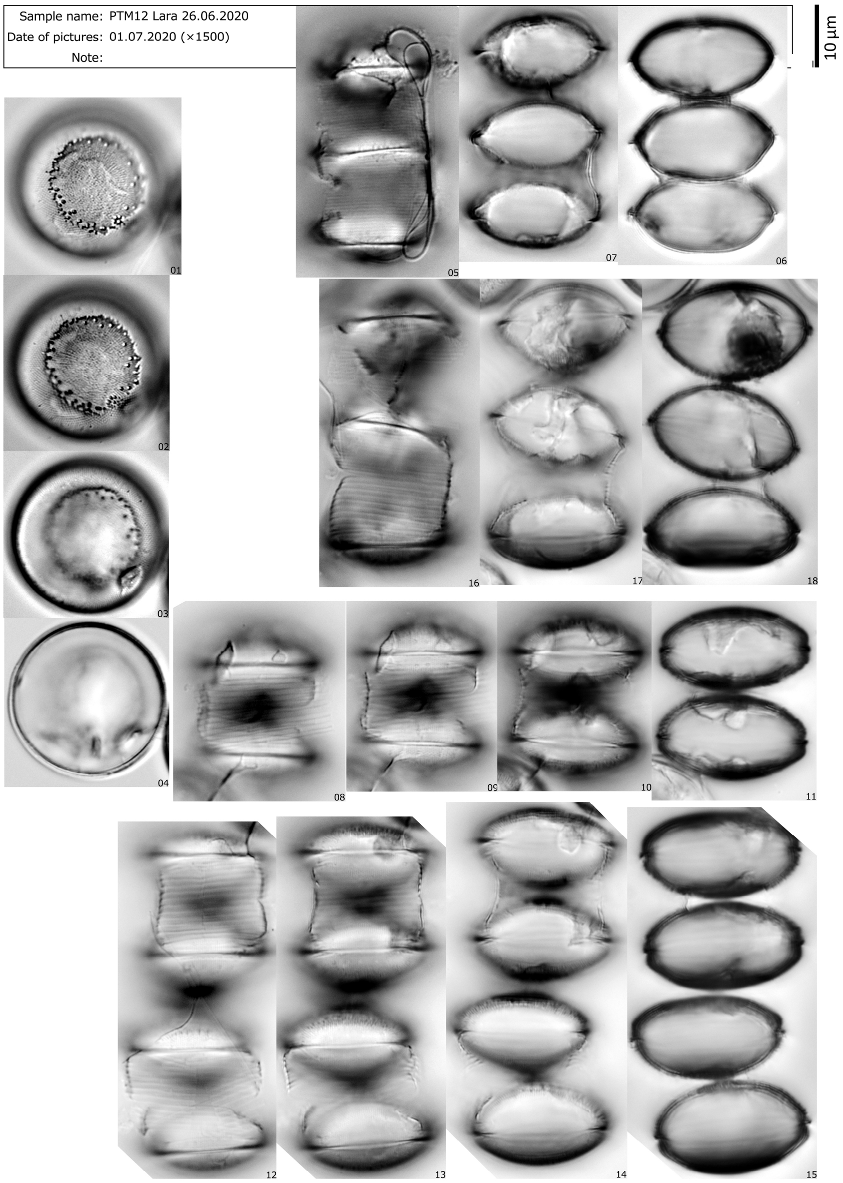

Figure S1. Identification plate with scanning electron microscopy images (by Kana Kuriyama) of the frustules of *Hyalodiscus scoticus*.

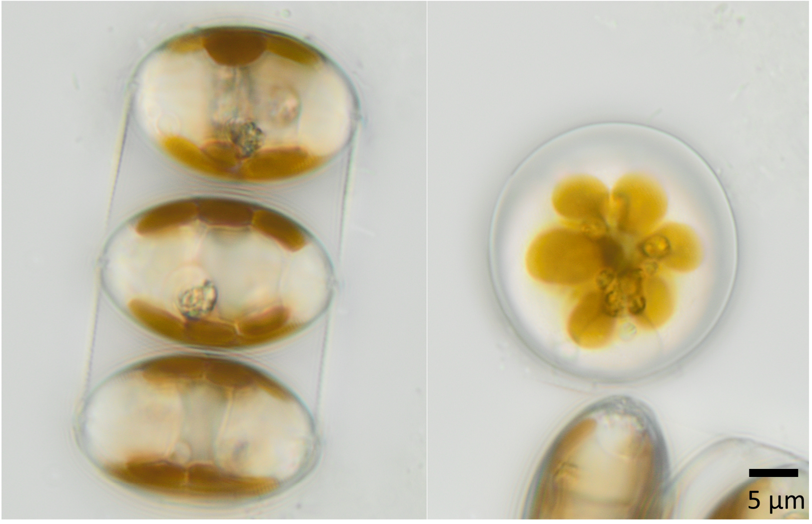

Figure S2. Light microscopy images of the vital cell *Hyalodiscus scoticus*.

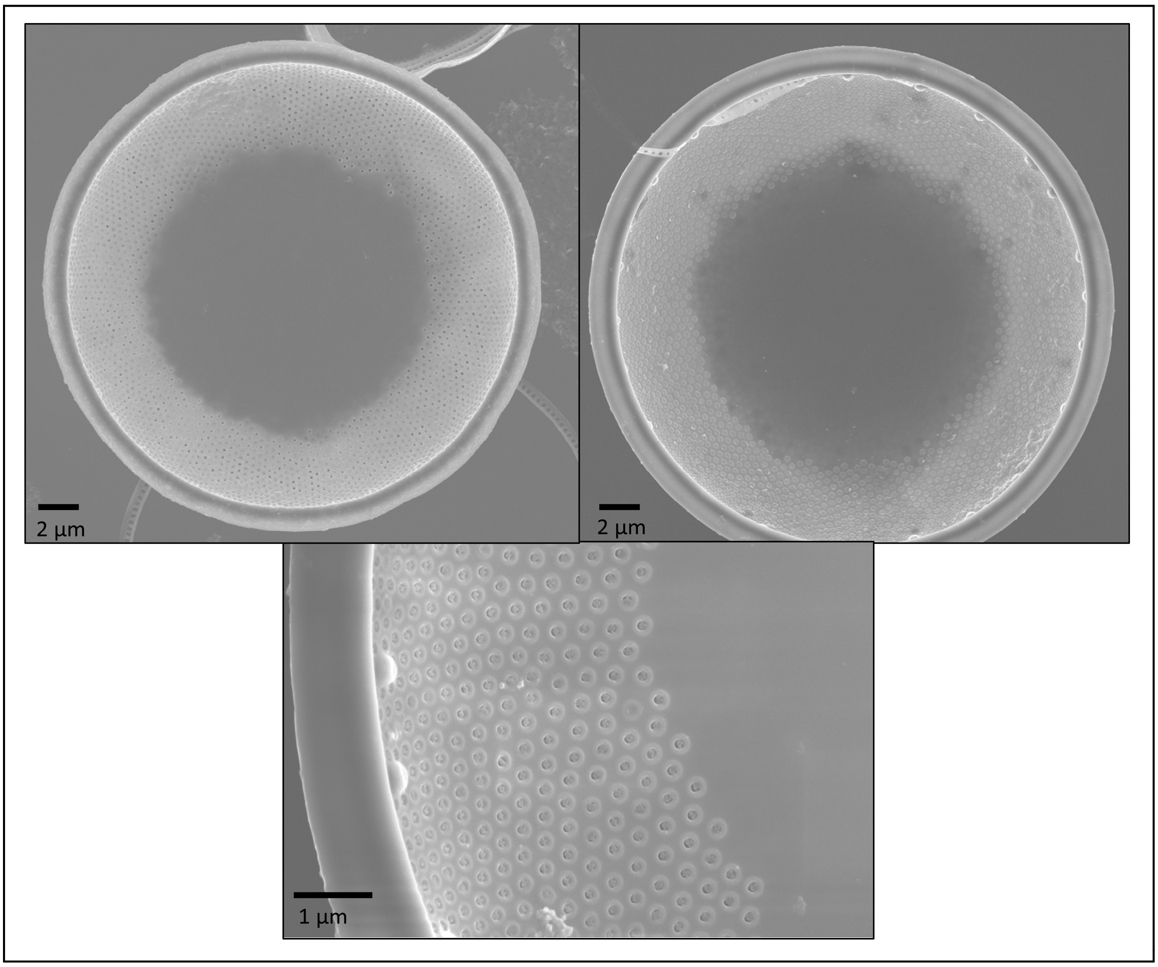

Figure S3. Scanning electron microscopy images of the frustules of *Hyalodiscus scoticus*.

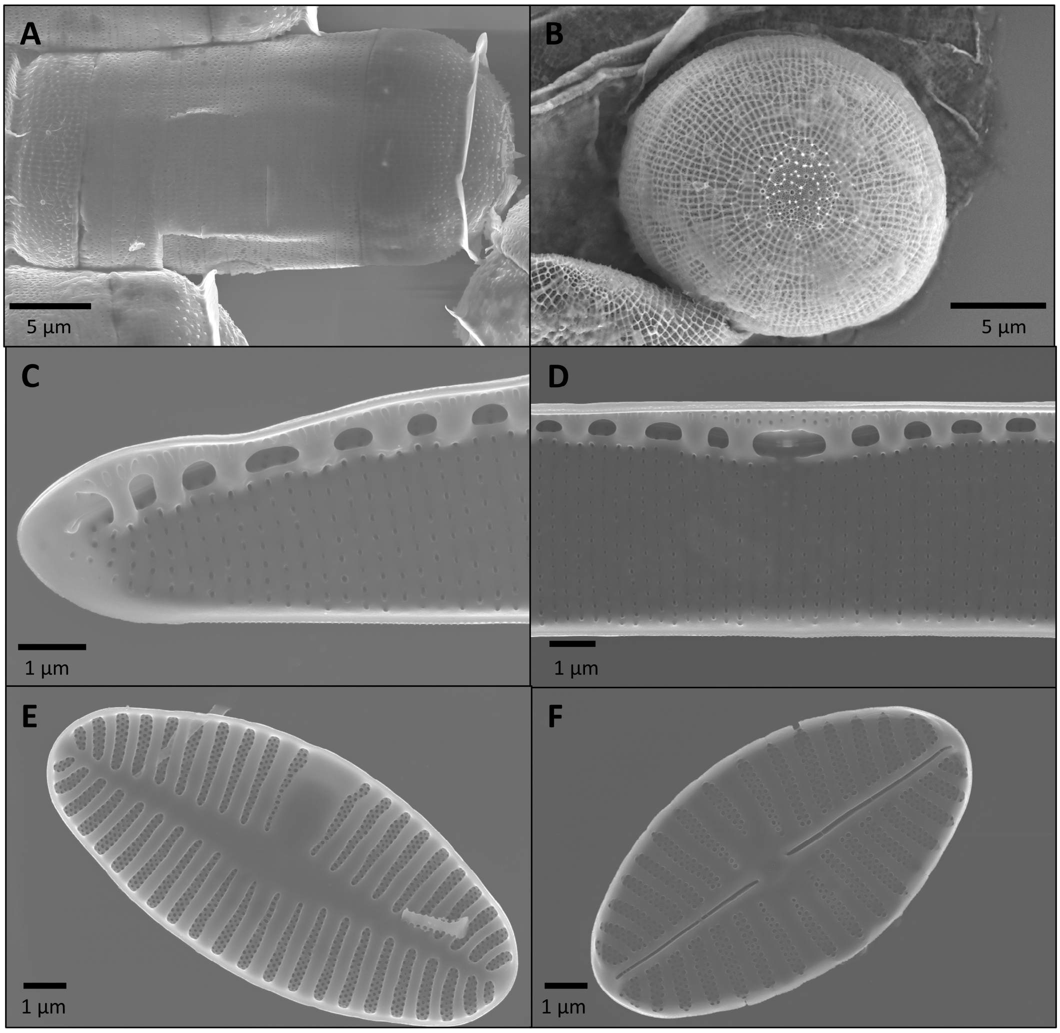

Figure S4. Scanning electron microscopy images of the frustules of (**A**), (**B**) *Melosira nummuloides*, (**C**), (**D**) *Nitzschia filiformis*, (**E**) *Planothidium* sp. (st. 1), (**F**) *Planothidium* sp. (st. 2).
