# Supplementary material for: Photosynthesis, Respiration, and Growth of Five Benthic Diatom Strains as a Function of Intermixing Processes of Coastal Peatlands with the Baltic Sea
Source: Microorganisms. 2022 Mar 30;10(4):749. doi: 10.3390/microorganisms10040749 (PMC9030513; doi:10.3390/microorganisms10040749)
Supplement: Supplementary file 1 [file microorganisms-10-00749-s001.zip › microorganisms-1645895-supplementary Tables.pdf]

Table S1 Confidence intervals for temperature-dependent photosynthetic and respirational rates of two benthic Baltic Sea diatom strains.

	2.5%	97.5%
***Melosira nummuloides* GM6**		
maximal photosynthetic rate	120.985	168.020
optimum photosynthetic temperature	4.327	13.716
maximum photosynthetic temperature	32.089	36.719
maximum respirational rate	-115.842	-85.969
optimum respirational temperature	33.163	36.112
maximum respirational temperature	41.404	47.804
***Melosira nummuloides* GM9**		
maximal photosynthetic rate	93.627	158.208
optimum photosynthetic temperature	-0.734	13.395
maximum photosynthetic temperature	30.529	36.984
maximum respirational rate	-77.547	-51.426
optimum respirational temperature	32.150	41.078
maximum respirational temperature	37.659	57.744
***Hyalodiscus cf. scoticus* GM6**		
maximal photosynthetic rate	48.900	99.277
optimum photosynthetic temperature	-0.340	8.654
maximum photosynthetic temperature	20.324	24.650
maximum respirational rate	-108.003	-73.185
optimum respirational temperature	31.596	35.049
maximum respirational temperature	40.474	46.239
***Hyalodiscus cf. scoticus* GM9**		
maximal photosynthetic rate	96.801	200.864
optimum photosynthetic temperature	11.297	25.130
maximum photosynthetic temperature	32.675	40.195
maximum respirational rate	-50.414	-34.560
optimum respirational temperature	31.651	35.768
maximum respirational temperature	40.474	49.443

Table S2 Confidence intervals for temperature-dependent photosynthetic and respirational rates of three benthic peatland diatom strains.

	2.5%	97.5%
***Planothidium* sp. (st. 2) GM6**		
maximal photosynthetic rate	50.027	64.185
optimum photosynthetic temperature	18.060	23.161
maximum photosynthetic temperature	38.377	42.978
maximum respirational rate	-51.693	-39.228
optimum respirational temperature	31.314	34.649
maximum respirational temperature	41.690	49.978
***Planothidium* sp. (st. 2) GM9**		
maximal photosynthetic rate	64.016	88.145
optimum photosynthetic temperature	16.519	23.643
maximum photosynthetic temperature	38.332	46.517
maximum respirational rate	-41.846	-29.968
optimum respirational temperature	31.433	36.698
maximum respirational temperature	40.586	53.530
***Nitzschia filiformis* GM6**		
maximal photosynthetic rate	63.125	112.760
optimum photosynthetic temperature	16.010	28.221
maximum photosynthetic temperature	34.995	55.693
maximum respirational rate	-86.450	-64.999
optimum respirational temperature	32.818	37.946
maximum respirational temperature	41.237	53.458
***Nitzschia filiformis* GM9**		
maximal photosynthetic rate	97.391	179.846
optimum photosynthetic temperature	10.977	25.748
maximum photosynthetic temperature	34.082	50.310
maximum respirational rate	-54.806	-44.706
optimum respirational temperature	32.763	34.856
maximum respirational temperature	42.381	46.782
***Planothidium* sp. (st. 1) GM6**		
maximal photosynthetic rate	85.206	101.016
optimum photosynthetic temperature	24.474	26.957
maximum photosynthetic temperature	38.861	40.533
maximum respirational rate	-51.439	-42.739
optimum respirational temperature	33.157	37.708
maximum respirational temperature	43.553	54.557
***Planothidium* sp. (st. 1) GM9**		
maximal photosynthetic rate	103.504	123.181
optimum photosynthetic temperature	24.846	27.405
maximum photosynthetic temperature	39.625	41.730
maximum respirational rate	-48.406	-40.454
optimum respirational temperature	33.080	35.243
maximum respirational temperature	43.048	48.022
